# Missouri

**Published:** 1896-10

**Authors:** 


*Missouri.* The serum-treatment of tuberculosis continues to be a subject of earnest investigation by Dr. Paul Paquin, formerly State Veterinarian of Missouri. For a number of years his experiments have consisted in the inoculation of the patient with antitoxin-serum obtained from horses. The serum is taken from the horses under a series of inoculations with the genuine tuberculosis, and when the animal has arrived at the point of immunity. He reports many patients relieved by the inoculations. Sufficient time has not elapsed to determine as to their permanent character (recovery?). He considers serum most effective when applied in the early stage of the disease.

